# Culture of fecal indicator bacteria from snail intestinal tubes as a tool for assessing the risk of *Opisthorchis viverrini* infection in *Bithynia* snail habitat

**DOI:** 10.1186/s13071-019-3313-2

**Published:** 2019-01-30

**Authors:** Courtney C. Nawrocki, Nadda Kiatsopit, Jutamas Namsanor, Paiboon Sithithaworn, Elizabeth J. Carlton

**Affiliations:** 1 Department of Environmental and Occupational Health, Colorado School of Public Health, University of Colorado, Anschutz Medical Campus, Aurora, CO USA; 2 Department of Parasitology, Faculty of Medicine, Khon Kaen University, Khon Kaen, Thailand; 3 Cholangiocarcinoma Research Institute, Faculty of Medicine, Khon Kaen University, Khon Kaen, Thailand

**Keywords:** *Opisthorchis viverrini*, Liver fluke, *Bithynia*, *E. coli*, Fecal indicator bacteria, Dissection

## Abstract

**Background:**

Like many trematodes of human health significance, the carcinogenic liver fluke, *Opisthorchis viverrini*, is spread *via* fecal contamination of snail habitat. Methods for assessing snail exposure to fecal waste can improve our ability to identify snail infection hotspots and potential sources of snail infections. We evaluated the feasibility of culturing fecal indicator bacteria from *Bithynia* snail intestinal tubes as a method for assessing snail exposure to fecal waste. Snails and water samples were collected from a site with a historically high prevalence of *O. viverrini* infected snails (“hotspot” site) and a site with historically no infected snails (“non-hotspot” site) on two sampling days. Snails were tested for *O. viverrini* and a stratified random sample of snails from each site was selected for intestinal tube removal and culture of gut contents for the fecal indicator bacteria, *Escherichia coli*. Water samples were tested for *E. coli* and nearby households were surveyed to assess sources of fecal contamination.

**Results:**

At the hotspot site, 26 of 2833 *Bithynia siamensis goniomphalos* snails were infected with *O. viverrini* compared to 0 of 1421 snails at the non-hotspot site. A total of 186 snails were dissected and cultured. *Escherichia coli* were detected in the guts of 20% of uninfected snails, 4% of *O. viverrini*-positive snails and 8% of snails not examined for cercarial infection at the hotspot site. Only one of 75 snails from the non-hotspot site was positive for *E. coli*. Accounting for sampling weights, snails at the hotspot site were more likely to have gut *E. coli* than snails from the non-hotspot site. The concentration of fecal indicator bacteria in surface water was higher at the hotspot *vs* non-hotspot site on only the first sampling day.

**Conclusions:**

Fecal indicator bacteria can be detected in the intestinal tubes of *Bithynia* snails. The presence of fecal indicator bacteria in *Bithynia* snail guts may indicate risk of *O. viverrini* infection in snail populations. This method has the potential to aid in identifying locations and time windows of peak snail infection risk and may be applicable to other trematodes of human-health significance.

**Electronic supplementary material:**

The online version of this article (10.1186/s13071-019-3313-2) contains supplementary material, which is available to authorized users.

## Background

*Opisthorchis viverrini* is a food-borne, trematode parasite that is endemic in Southeast Asia including parts of Thailand, Cambodia, Vietnam and Lao PDR. Approximately 10 million people are infected, primarily rural farmers who consume raw or undercooked cyprinid fish, which harbor the parasite [1]. *Opisthorchis viverrini* causes cholangiocarcinoma (CCA), a highly fatal cancer in endemic regions, and heavy infection is associated with a range of hepatobiliary complications [2, 3]. Due to its public health impact, opisthorchiasis and CCA have received considerable attention from the scientific community [4–6]. Recent progress has been made, particularly in the areas of the epidemiology of human infection and pathogenesis [7]. It is also known that *O. viverrini* is a species complex consisting of at least two cryptic species in Thailand and Lao PDR which are related to biological characteristics of the parasites [8–10]. Recently *O. viverrini* from Sakon Nakhon, in northeast Thailand, were discovered as another cryptic species distinct from other isolates in Thailand and Lao PDR [11]. The snail intermediate host, *Bithynia siamensis goniomphalos*, has been shown to contain distinct cryptic species [12]. However, less is known about the risk factors for *O. viverrini* infections in these snails, and this information may be crucial to the long-term control and elimination of this disease.

We are interested in methods for assessing snail exposure to fecal waste as a means of identifying snail habitat that is likely playing a key role in disease transmission. Like many trematode parasites of human health significance including schistosomes, *Fasciola* spp., *Paragonimus* spp. and *Clonorchis sinensis*, eggs of *O. viverrini* are shed in the stool of humans and other competent mammalian hosts and the parasite requires a snail host to complete part of the developmental process before reaching a stage infectious to humans (*O. viverrini* must additionally infect cyprinid fish). Fecal contamination of snail habitat is a necessary condition for transmission of these parasites. Snail infection prevalence is often below 1% in endemic areas [13], although members of our group have reported on snail infection hotspots where the prevalence of *O. viverrini* infection in *Bithynia* snails was as high as 8% [14]. This spatial heterogeneity in snail infections raises interesting questions about the causes of such patterns and underscores the need for tools to distinguish high and low risk areas for snail infection. Methods for assessing snail exposure to fecal waste can improve our ability to identify likely snail infection hotspots, and better define the conditions under which humans and other mammals contribute to snail infections and thus propagate the *O. viverrini* transmission cycle.

One method for assessing snail exposure is through measurement of fecal indicator bacteria in surface water in and around snail habitat. Fecal indicator bacteria such as *Escherichia coli* are appropriate indicators of fecal contamination due to their presence in the typical gut microflora of humans and other animals. Kaewkes et al. [15] found that concentrations of fecal indicator bacteria in water collected from snail habitat can be linked to temporal and spatial patterns of snail infections. Recent studies have used this approach to define pathways by which poor sanitation [16, 17], landscape characteristics [17] and snail habitat type [18] impact fecal contamination of *Bithynia* snail habitat. However, these recent studies have found few or no infected snails at their sampling sites [16–18], which makes it challenging to link these potential infection pathways to actual snail infections.

Another potential method for assessing snail exposure is through examination of snail gut flora. Snail gut dissection has been used to study microflora in terrestrial edible snails *Cornu aspersum* and *Helix pomatia* and the aquatic snail *Pomacea canaliculata* [19–22]. Dissection and culture of gut contents proves an effective way of characterizing gut bacteria when done aseptically. This method has the potential to be used to measure fecal indicator bacteria in the guts of *Bithynia* snails. *Bithynia* snails become infected with *O. viverrini* when they eat the parasite egg, which is shed in the stool of definitive hosts. Thus, culture of gut contents has the potential to provide a direct measure of snail exposure to fecal waste.

The purpose of this study was to evaluate the feasibility of snail gut dissection and culture of fecal indicator bacteria as a tool for assessing the risk of *O. viverrini* infections in *Bithynia* snail habitat. We field-tested the method in two locations: a suspected snail infection hotspot and an area where snail infection risk was expected to be low, and validated these classifications through snail infection testing. We tested water samples for fecal indicator bacteria in parallel, allowing us to compare the methods. To our knowledge, this is the first reported application of culture of snail gut fecal indicator bacteria in the context of *O. viverrini* surveillance.

## Methods

This study was conducted in Sakon Nakhon Province in northeastern Thailand, where *O. viverrini* is endemic. Two distinct study locations were selected, one where the research team had consistently found infected snails in the past (the “hotspot site”) and another where they had not (the “non-hotspot site”), in order to allow comparison of snail exposure methods in sites with high *vs* low risk of *O. viverrini* infection. Both sites are rice paddies, located approximately 39 km apart. The low risk site is located in Phanna Nikhom District, Na Nai sub-district. This site is relatively isolated: there are no major villages nearby, the road to the site had little traffic and no bovines were observed during sampling. The hotspot site was located in Phang Khon District, an area where snail infection hotspots had previously been documented [14]. This site is in the Hai Yong sub-district. In contrast to the first site, the hotspot site is adjacent to a nearby village, with the closest households approximately 20 m from the rice paddy. The rice paddy is also next to a main road with regular car and foot traffic and bovines were observed nearby during sampling.

### Snail collection

*Bithynia siamensis goniomphalos* snails were collected by handpicking and dredging with a scoop from the two study locations on two days in summer of 2017, 11 days apart (21 June 2017 and 2 July 2017). Following collection, snails were kept alive, separated by site and transported to the laboratory in the Khon Kaen University Department of Parasitology. Snails were fed sterilized ivy gourd once daily.

### Determination of snail infection *via* cercarial shedding method

Snails were analyzed for infection *via* the cercarial shedding method [23] one day following snail collection. Each snail was placed in a separate plastic cup (3 cm in diameter, 2.5 cm in height) with 5 ml dechlorinated water and covered with a lid with small holes in it to allow snails to breathe and keep them contained. Snails were exposed to artificial light (1200 1x) for 5 h during the day at room temperature (25 ± 2 °C) to stimulate the cercarial shedding process. Each individual cup was then inspected for the presence of cercariae using a stereomicroscope. Cercariae were morphologically identified using a high-magnification compound microscope. All suspected cercarial infections were confirmed by a second technician. Snails were separated into groups of uninfected, *O. viverrini*-positive and non-*O. viverrini* infection-positive, and stored in plastic containers with a mesh top until the following day.

### Snail dissection and culture of fecal indicator bacteria from snail intestinal tubes

Intestinal tubes were dissected, removed and cultured for *E. coli* from snails obtained from each site two days after sample collection. Prior to dissection, the shells were wiped with standard physiological solution (0.85% NaCl) and snails were aseptically removed from their shells in sterile Petri dishes while carefully avoiding contact with the outer surface. A stereomicroscope was used to dissect all snails *in vivo* under aerobic conditions as described by Koleva et al. [24]. The intestinal tract, from esophagus to rectum, was removed and rinsed three separate times in sterile saline solution to avoid contamination from other snail tissues. Each intestinal tube sample was then diluted with 1 ml of physiological solution in an Eppendorf tube and vortexed for several seconds to release the contents of the tube. A 1 ml aliquot of solution was then plated onto *E.coli*/Coliform Count Petrifilms (3M, St. Paul, MN, USA) and incubated in aerobic conditions for 24 h at 37 ± 2 °C. Immediately following incubation, two technicians independently counted all blue colonies with gas bubbles as *E. coli* colony-forming units (CFUs) per the manufacturer’s instructions [25].

Four types of snails were dissected in order to evaluate differences in *E. coli* culture results by snail infection status. From each site, 30 snails negative for any cercarial infection, 30 snails positive for non-*O. viverrini* cercarial infections and all *O. viverrini*-positive snails were dissected. Additionally, a sample of snails from each site were not tested for infection and were dissected without undergoing the cercarial shedding process in order to evaluate the possibility that the snail shedding process may affect gut bacteria. Like the shedding snails, these snails were dissected two days after sample collection.

### Water collection and culture of fecal indicator bacteria

Water samples were collected from both sites on the same two days as snail collection in shallow areas (< 0.3 m deep). At each sampling location, water samples were collected in sterile 18 oz. (532 ml) Whirl-Pak bags by dipping the bag just under the surface. Samples were then labeled, placed on ice and immediately transported back to the laboratory at the Khon Kaen University Department of Parasitology. On the first day of sampling, one water sample was collected from each site. In order to assess within-site variability in *E. coli* concentrations in water, water samples were collected from five distinct locations at each site on the second day of sampling.

Water samples were cultured immediately upon arrival at the laboratory. Five milliliters from each water sample was cultured. Samples were vortexed and 1 ml aliquots were plated on *E.coli*/Coliform Count Petrifilms (3M). As above, the films were incubated in aerobic conditions for 24 h at 37 ± 2 °C, blue colonies with gas bubbles were counted as *E. coli* CFUs immediately following the incubation period and confirmed by a second reader. If *E. coli* colonies were too numerous to count, CFUs within one of the 20 squares that visually divide the Petrifilm were counted and this total was multiplied by 20 to estimate the CFU/ml concentration for the replicate, per the manufacturer’s instructions [25]. The *E. coli* concentration in CFU/ml at each location was estimated by summing the number of *E. coli* CFUs on each Petrifilm, then dividing by five. Based on the high concentrations of *E. coli* cultured from the first sampling day, samples from the hotspot site on the second sampling day were diluted by a factor of 1:10.

### Household surveys

In order to assess potential sources of fecal contamination, structured questionnaires were administered at ten households located in the area surrounding the hotspot site. Participants provided written informed consent and surveys were only administered if the individual was over the age of 18. Questions were asked about septic tank construction, sanitation practices, domestic animals and pets.

### Statistical analysis

We tested the hypothesis that snail cercarial infections, the presence of *E. coli* in snail guts, and the concentration of *E. coli* in water differed between hotspot and non-hotspot sites. The Chi-square test for independence was used for binary measures, Fisher’s exact test was used when expected cell counts were less than five, and the non-parametric Wilcoxon rank sum test was used for continuous measures. Because we sampled on two different sampling days, essentially drawing two samples from each site, statistical tests were conducted for each sampling day separately, as well as using data aggregated across both days. We compared the proportion of snails with gut *E. coli* between uninfected snails and snails not examined for cercarial infection, and by snail infection type using Fisher’s exact test. Stata 15 statistical software was used for statistical analysis [26]. All tests of statistical significance were tested at α = 0.05.

We estimated the expected number of snails with detectable *E. coli* in the intestinal tube if we randomly sampled 100 snails at each site by calculating a weighted average that accounted for the proportion of snails with each type of infection (no infection, *O. viverrini* infection, other cercarial infection) and the proportion of snails of each infection type with detectable *E. coli*. In order to account for uncertainty, estimates were generated separately using the results from each sampling day. As a sensitivity analysis, we generated a second set of estimates using the proportion of *E. coli* detected in snails that were not tested for cercarial infection, as these snails represented a random sample of the snails collected each day, albeit a small sample.

## Results

### Snail infection

Of the 1421 snails collected from the non-hotspot site, zero tested positive for *O. viverrini* (Table 1). In contrast, 26 of 2833 snails from the hotspot site (0.9%) tested positive for *O. viverrini*. The prevalence of *O. viverrini* infections in *B. s. goniomphalos* snails at the hotspot site was significantly higher than at the non-hotspot site on the second day of sampling (Chi-square test, *χ*^2^ = 11.28, *df* = 1, *P* = 0.001) and overall (Chi-square test, *χ*^2^ = 13.12, *df* = 1, *P* < 0.001), but not on the first day (Fisher’s exact test, *P* = 0.071).

**Table 1:** Cercarial infections in *Bithynia siamensis goniomphalos* snails collected from two sampling locations

Snails by infection status	Hotspot			Non-hotspot		
	Day 1, *n* (%)	Day 2, *n* (%)	Total, *n* (%)	Day 1, *n* (%)	Day 2, *n* (%)	Total, *n* (%)
Snails tested	1676	1157	2833	612	809	1421
*O. viverrini*-positive	10 (0.6)	16 (1.4)	26 (0.9)	0	0	0
Other trematode infections	26 (1.6)	75 (6.5)	101 (3.6)	47 (7.7)	118 (14.6)	165 (11.6)
Xiphidiocercariae	25 (1.5)	73 (6.3)	98 (3.5)	44 (7.2)	100 (12.4)	144 (10.1)
Echinostome cercariae	1 (0.1)	1 (0.1)	2 (0.1)	0	3 (0.4)	3 (0.2)
Cystophorous cercariae	0	1 (0.1)	1 (0.04)	1 (0.2)	3 (0.4)	4 (0.3)
Amphistome cercariae	0	0	0	2 (0.3)	1 (0.1)	3 (0.2)
Parapleurolophocercous cercariae	0	0	0	0	11 (1.4)	11 (0.8)
Mixed infection^a^	0	0	0	0	1 (0.1)	1 (0.1)

^a^One snail was infected with xiphidiocercariae and cystophorous cercariae

More snails tested positive for other cercarial infections at the non-hotspot site (11.6%) compared to the hotspot site (3.6%), a finding unlikely due to chance (Chi-square test, *χ*^2^ = 104.52, *df* = 1, *P* < 0.001 comparing overall prevalence between sites; Chi-square test, *χ*^2^ = 54.51, *df* = 1, *P* < 0.001 on day 1; Chi-square test, *χ*^2^ = 35.31, *df* = 1, *P* < 0.001 on day 2). Xiphidiocercariae infections were the most commonly seen infections at both sites.

### *Escherichia coli* concentrations in snail intestinal tubes

**Fig. 1:**
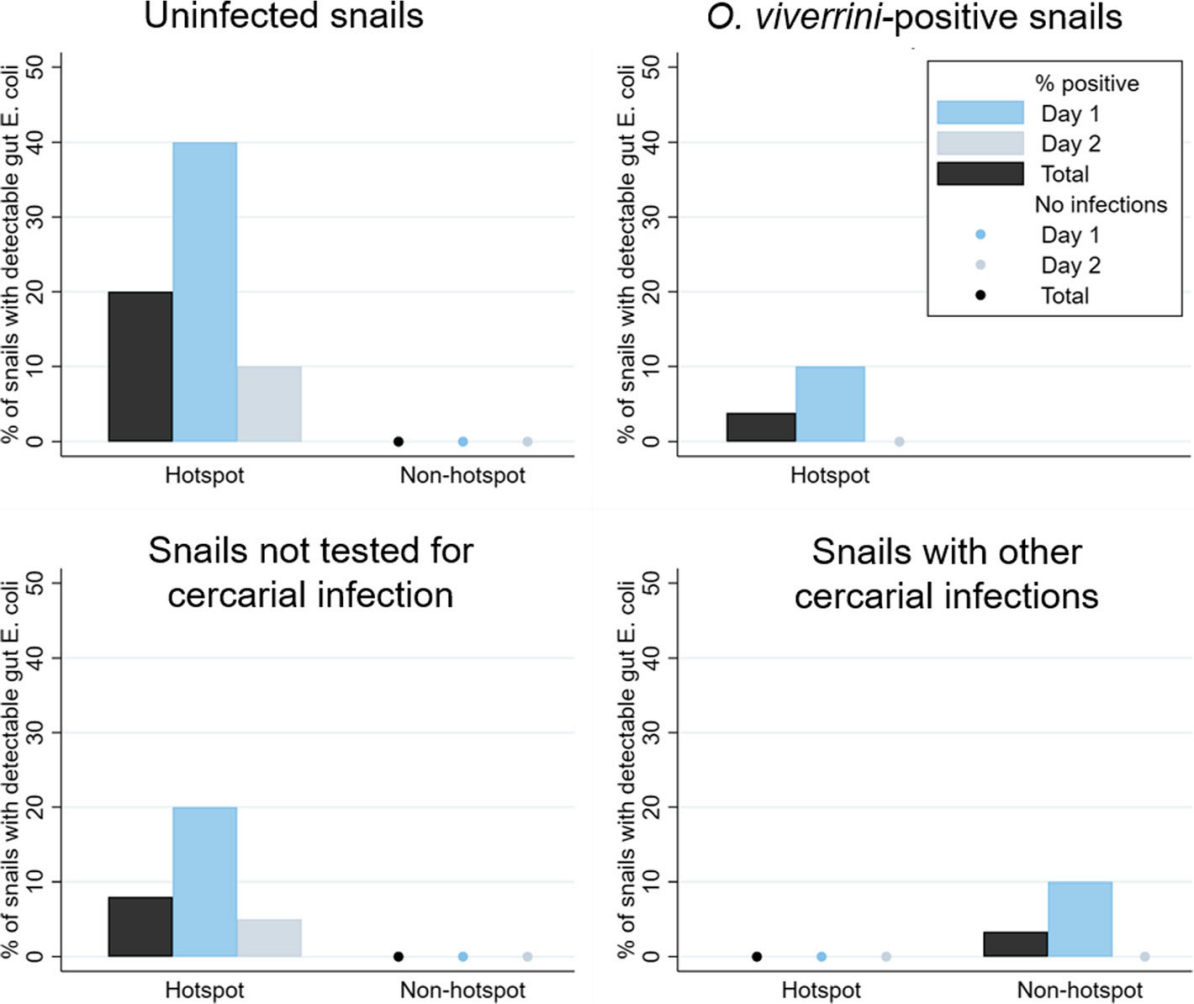
The percent of snails with detectable gut *E. coli* by location, shown for uninfected snails (top left), snails not tested for trematode infection (bottom left), snails infected with *O. viverrini* (top right), and snails with other trematode infections (bottom right). The only *O. viverrini*-positive snails cultured for gut *E. coli* were from the hotspot site as no *O. viverrini*-positive snails were found at the non-hotspot site

*Escherichia coli* was cultured from the intestinal tubes of 6 of 30 uninfected snails from the hotspot site (20%) and 0 of 30 uninfected snails from the non-hotspot site (Fig. 1 and Additional file 1: Table S1). The concentration of *E. coli* in *E. coli-*positive snails was generally low, ranging between 1–8 CFUs. The probability of detecting *E. coli* in the intestinal tubes of uninfected snails was higher in the hotspot *vs* non-hotspot site (Fisher’s exact test, *P* = 0.024) and these patterns were consistent on each day of sampling, although the differences each day may be due to chance (Fisher’s exact test, day 1: *P* = 0.087; day 2: *P* = 0.487).

The snail shedding process did not appear to impact the presence of *E. coli* in snail guts. We found *E. coli* in the guts of 20% of uninfected snails *vs* 8% of snails not examined for cercarial infection at the hotspot site (Fisher’s exact test, *P* = 0.269), and in 0% of both types of snails at the non-hotspot site.

The relationship between the presence and type of cercarial infections in snails and detection of gut *E. coli* varied by site. At the hotspot site, uninfected snails were most likely to have detectable gut *E. coli* (Fisher’s exact test, *P* = 0.009). At the non-hotspot site, the only snail with detectable gut *E. coli* was a snail with a non-*O. viverrini* cercarial infection, although this small difference in the detection of gut *E. coli* in snails with cercarial infections *vs* uninfected snails was likely due to chance (Fisher’s exact test, *P* = 1.000).

Overall, *E. coli* were more likely to be detected in snails from the hotspot site *vs* the non-hotspot site. The expected number of snails with detectable *E. coli* if 100 were randomly sampled was 19 at the hotspot site (range 9–39), compared to 0.4 at the non-hotspot (range 0–0.8). Estimates generated using data from non-shed snails were similar: we estimated 8 of 100 snails with detectable *E. coli* at the hotspot site (range 5–20) and 0 snails with detectable *E. coli* at the non-hotspot site.

### *Escherichia coli* concentrations in water samples

On the first sampling day, the *E. coli* concentration in water at the hotspot site was much higher than the non-hotspot site (1864 *vs* 1.2 CFU/ml). On the second day of sampling, *E. coli* concentrations in water samples were similar at both sites: the hotspot site averaged 0.8 ± 1.1 SD CFU/ml and the non-hotspot site averaged 1.4 ± 1.0 CFU/ml (Wilcoxon rank-sum test, *Z*= -1.26, *P* = 0.207). The concentrations of *E. coli* were similar across the five locations sampled at each site on the second day, ranging between 0–2.6 CFU/ml at the hotspot site and 0.6–3.2 CFU/ml at the non-hotspot site.

### Possible sources of fecal contamination

Of the 10 individuals interviewed near the hotspot site with the household questionnaire, all ten reported that they or others in their household consume fish dishes that are typically eaten raw. Six of these individuals reported that they only consume these dishes cooked, while four reported consuming the dishes both cooked and raw. All interviewees reported having a circular, cemented, conventional septic tank system at their household; however, only one person reported that their septic tank was fully cemented on the top, sides and bottom, while the rest reported that their septic tank was only cemented on the tops and sides. Two individuals reported that their toilet or septic tank has flooded or overflowed in the past year due to heavy rains. All 10 households reported owning at least one domestic pet, including cats (3 households) and dogs (10 households). No households reported using septic tank waste to fertilize agricultural fields.

## Discussion

Our findings demonstrate that the culture of *Bithynia* snail gut contents for the fecal indicator bacteria *E. coli* is feasible and may serve as a useful tool for monitoring fecal contamination around *Bithynia* snail habitats. We found that *E. coli* are present in the intestinal tubes of *B. s. goniomphalos* snails in a region where *O. viverrini* is endemic, and were consistently detected at a site where *O. viverrini*-infected snails were found. In contrast, we found only one snail with detectable gut *E. coli* at a site where no *O. viverrini*-infected snails were found. This method appears robust to the cercarial shedding process, suggesting it could be used alone or in combination with snail cercarial infection testing. Because snails are exposed to *O. viverrini* through consumption of its eggs, measuring snail gut fecal indicator bacteria provides a direct measure of snail exposure to fecal waste, a condition necessary for *O. viverrini* transmission.

The dissection and culture of snail gut contents for fecal indicator bacteria provides a new tool for identifying snail habitat that is contributing to the *O. viverrini* transmission cycle. The most widely used method for characterizing transmission risk in snail habitat has been and remains monitoring *O. viverrini* infections in snails. This approach has provided key information on the spatial heterogeneity and temporal patterns of snail infection (e.g. [14, 27]) and is a definitive indicator of parasite presence, if not a perfect indicator of parasite absence. However, the prevalence of *O. viverrini* and other trematode infections in snails is often below 1% in endemic areas, necessitating testing thousands of snails at each surveillance site. In fact, as opisthorchiasis control measures have been implemented, many groups have struggled to find infected snails in areas where human infections are present (e.g. [16, 18]), an experience we have also had with the trematode *S. japonicum* [28].

Monitoring fecal indicator bacteria in and around snail habitat offers the potential to characterize snail infection risk based on fecal input into snail habitat. Recent studies have related fecal indicator bacteria concentrations in surface water in and around snail habitat to potential sources of fecal contamination, such as septic tanks [16, 17], and to temporal patterns of snail infections [15]. Testing water for fecal indicator bacteria is relatively inexpensive, does not require sophisticated laboratory equipment and requires far fewer samples than snail infection testing. However, concentrations of *E. coli* in water can be highly variable over short time-scales, due in part to rainfall [29]. We found *E. coli* concentrations in surface water varied by three orders of magnitude within a two week period at the hotspot site. This is likely due to the fact that a heavy rainfall event preceded the second collection day, diluting the concentration of fecal indicator bacteria. Assessment of snail gut fecal indicator bacteria, while more labor-intensive than water monitoring, may provide a more stable measure of fecal contamination relevant to *O. viverrini* transmission than water sampling.

We see several potential applications of snail gut culture for fecal indicator bacteria in the context of *O. viverrini*. First, we suspect that rainfall plays a key role in the mobilization and transport of *O. viverrini* eggs, present in fecal waste, to snail habitat [30, 31]. Coupled longitudinal sampling of fecal indicator bacteria in water and snail gut contents can help shed light on the pulses of fecal contaminants that lead to direct snail exposure to fecal waste. Secondly, it is possible that monitoring fecal indicator bacteria can provide an early warning of snail infection risk. The cercarial shedding method identifies snails with patent infections. Snails shedding *O. viverrini* cercariae reflect exposure to fecal waste containing *O. viverrini* eggs two or more months prior, due to the approximately two month lag between infection and cercarial shedding. Monitoring fecal indicator bacteria capitalizes on this lag, providing an early indicator of potential snail infections in snail habitat, which could trigger intervention measures such as snail control. Notably, we found *O. viverrini*-infected snails were not more likely to have detectable *E. coli* in their guts compared to uninfected snails, suggesting uninfected (or pre-patent) snails may be an appropriate surveillance target for monitoring potential exposure of snails to fecal waste.

Thirdly, our method of snail gut culture could be adapted to leverage advances in microbial source tracking in order to assess the contribution of distinct mammalian hosts to fecal contamination of snail habitat (e.g. [32–34]). *Escherichia coli* are present in the guts of warm-blooded animals [35] but *O. viverrini* is capable of infecting only fish-eating mammals including humans, cats and dogs [36]. The use of *E. coli* as an indicator of fecal contamination is inherently noisy in that it may indicate fecal contamination from definitive hosts such as humans, as well as non-reservoir animals such as bovines. If humans are the only necessary host in the *O. viverrini* life-cycle as a recent theoretical study concluded [37], focusing on human-specific indicators of fecal contamination can remove noise from animals that are not potential sources of infection, providing a more precise measure of snail exposure. It also has the potential to shed light on the role of non-human definitive hosts in the transmission cycle, a question of long-standing interest [36, 37].

### Limitations and caveats

This study was designed to evaluate the feasibility of snail gut dissection and therefore sample sizes were small and sampling was limited to the rainy season. Replication of the above methods over a larger spatial area and across different seasons and rice development stages, in combination with potential source mapping, is needed to assess how this method generalizes to other *O. viverrini* populations and *Bithynia* species as a tool for discriminating snail infection hotspots and peak windows of snail infection risk. Additionally, a controlled laboratory study to assess the time between *E. coli* exposure and colonization, the infectious dose of *E. coli* for colonization, the length of time *E. coli* survives in the guts of snails, and the impact of *E. coli* on snail survival would be beneficial in estimating potential exposure windows and defining optimal sample processing times following collection. Lacking this information, we are unable to estimate the fraction of snails that may have been positive for *E. coli* at the time of collection, but negative at the time of dissection. Similarly, some snails did not survive prior to shedding and dissection and therefore did not undergo those processes. To the extent this occurred, our estimates of the presence of *E. coli* in snail guts represent lower bound estimates.

Due to the widespread presence of *E. coli*, use of this method requires measures to preserve original *E. coli* concentrations in the samples and limit potential for contamination. To this end, we kept snails separate during the shedding process: each snail was housed in an individual container, snails were rinsed in saline at each step as they underwent the dissection process to reduce the risk of cross-contamination, and care was taken to not contaminate snail tissue with bacteria from the surface of the snail shell.

## Conclusions

The culture of *Bithynia* snail gut contents for the fecal indicator bacteria *E. coli* is feasible and may serve as a useful tool for identifying snail habitat that is contributing to the transmission of *O. viverrini*. Because snails are exposed to *O. viverrini* through consumption of parasite eggs, measuring snail gut fecal indicator bacteria provides a direct measure of snail exposure to fecal waste. This method has the potential to be used to characterize key sources of *O. viverrini* eggs in snail habitat as well as to clarify the relationships between fecal waste contamination, rainfall and snail infection with the long term goal of being able to provide an actionable early warning of snail infection risk.

## Additional file

Additional file 1: **Table S1.** The presence and concentration of *E. coli* cultured from dissected snails at two sampling locations. (DOCX 16 kb)

## Abbreviations

CCA: Cholangiocarcinoma
CFUs: Colony-forming units
