# Supplementary material for: Culture of fecal indicator bacteria from snail intestinal tubes as a tool for assessing the risk of Opisthorchis viverrini infection in Bithynia snail habitat
Source: Parasit Vectors. 2019 Jan 30;12:66. doi: 10.1186/s13071-019-3313-2 (PMC6354346; doi:10.1186/s13071-019-3313-2)
Supplement: Supplementary file 1 — Table S1. The presence and concentration of E. coli cultured from dissected snails at two sampling locations. (DOCX 16 kb) [file 13071_2019_3313_MOESM1_ESM.docx]

**Additional file 1: Table S1.** The presence and concentration of *E. coli* cultured from dissected snails at two sampling locations.

|  | **Hotspot** | | |  | **Non-hotspot** | | |
| --- | --- | --- | --- | --- | --- | --- | --- |
|  | **Day 1** | **Day 2** | **Total** |  | **Day 1** | **Day 2** | **Total** |
| Uninfected snails |  |  |  |  |  |  |  |
| Dissected | 10 | 20 | 30 |  | 10 | 20 | 30 |
| Positive for gut E. coli (%) | 4 (40) | 2 (10) | 6 (20) |  | 0 (0) | 0 (0) | 0 (0) |
| Median CFU/1mL (Range)^a^ | 4 (1-8) | 1 (N/A) | 2 (1-8) |  | 0 (0) | 0 (0) | 0 (0) |
| O. viverrini positive snails^b^ |  |  |  |  |  |  |  |
| Dissected | 10 | 16 | 26 |  | 0 | 0 | 0 |
| Positive for gut E. coli (%) | 1 (10) | 0 (0) | 1 (4) |  | - | - | - |
| Median CFU/1mL (Range) ^a^ | 2 (N/A) | 0 (0) | 2 (N/A) |  | - | - | - |
| Other trematode positive snails |  |  |  |  |  |  |  |
| Dissected | 10 | 20 | 30 |  | 10 | 20 | 30 |
| Positive for gut E. coli (%) | 0 (0) | 0 (0) | 0 (0) |  | 1 (10) | 0 (0) | 1 (3) |
| Median CFU/1mL (Range) ^a^ | 0 (0) | 0 (0) | 0 (0) |  | 5 (N/A) | 0 (0) | 5 (N/A) |
| Snails not tested for cercarial infection |  |  |  |  |  |  |  |
| Dissected | 5 | 20 | 25 |  | 5 | 10 | 15 |
| Positive for gut E. coli (%) | 1 (20) | 1 (5) | 2 (8) |  | 0 (0) | 0 (0) | 0 (0) |
| Median CFU/1mL (Range) ^a^ | 2 (N/A) | 1 (N/A) | 1.5 (1-2) |  | 0 (0) | 0 (0) | 0 (0) |
| Total snails dissected | 35 | 76 | 111 |  | 25 | 50 | 75 |
| Total snails positive for gut E. coli (%) | 6 (17) | 3 (4) | 9 (8) |  | 1 (4) | 0 (0) | 1 (1.33) |

N/A= Not applicable

*^a^*Median and range presented for positive samples.

*^b^*No *O. viverrini* positive snails were found at the non-hotspot site.
